# Characterization of two novel HIV-1 second-generation recombinants (CRF01_AE/CRF07_BC) identified in Hebei Province, China

**DOI:** 10.3389/fmicb.2023.1159928

**Published:** 2023-05-03

**Authors:** Xuegang Yang, Na Zhao, Miaomiao Su, Juan Meng, Jian Du, Weina An, Haoxi Shi, Weiguang Fan

**Affiliations:** ^1^Infection Division, The People’s Hospital of Baoding, Baoding, Hebei, China; ^2^Clinical Laboratory, The People’s Hospital of Baoding, Baoding, Hebei, China

**Keywords:** HIV, circulating recombinant forms, near full-length genome, unique recombination forms, MSM

## Abstract

**Introduction:**

The unique recombinant forms (URFs) of HIV-1 consist of a mixture of subtypes, and each URF has a unique breakpoint. In this study, we identified the near fulllength genome (NFLG) sequences of two novel HIV-1 URFs (Sample ID: BDD034A and BDL060) isolated during HIV-1 molecular surveillance in 2022 in Baoding city, Hebei Province, China.

**Methods:**

The two sequences were aligned with subtype reference sequences and CRFs from China using MAFFT v7.0, and the alignments were adjusted manually using BioEdit (v7.2.5.0). Phylogenetic and subregion trees were constructed using MEGA11 with the neighbor-joining (N-J) method. Recombination breakpoints were identified by SimPlot (v3.5.1) based on Bootscan analyses.

**Results:**

Recombinant breakpoint analysis revealed that the NFLGs of BDD034A and BDL060 were composed of CRF01_AE and CRF07_BC, containing seven segments, respectively. For BDD034A, three CRF01_AE fragments were inserted into the CRF07_BC main framework, whereas for BDL060, three CRF07_BC fragments were inserted into the CRF01_AE main framework.

**Discussion:**

The emergence of the CRF01_AE/CRF07_BC recombinant strains indicates that HIV-1 co-infection is common. The increasing genetic complexity of the HIV-1 epidemic in China warrants continued investigation.

## Introduction

The high rates of mutation, recombination and replication that are characteristic of HIV-1 mean that new circulating recombinant forms (CRFs) and unique recombinant forms (URFs) are constantly emerging (Hemelaar, 2012; Bbosa et al., 2019). To date, 132 CRFs, as well as numerous URFs, have been registered in the Los Alamos National Laboratory HIV database.^1^ In China, CRF01_AE and CRF07_BC are the predominant intersubtype recombinant forms of HIV-1, with recombinant forms between these two subtypes emerging, most prevalently among men who have sex with men (MSM), in recent years (He et al., 2012; Li et al., 2016; Yin et al., 2019). The continuous emergence of new recombinant forms has brought new challenges to the monitoring, treatment and prevention of HIV infection. Hebei is a northern province of China with a low HIV prevalence (Lu et al., 2017); however, over the last 10 years, the number of individuals infected with HIV-1 through sexual contact has reached 98.9%, among which 77.5% of cases are in MSM, with CRF01_AE (49.6%), CRF07_BC (29.7%) and B subtype (13.0%) being the three main genotypes (Lu et al., 2020). The co-circulation of CRF01_AE and CRF07_BC strains and the dual infection of these strains in the sexually-active population has increased the generation of inter-subtype recombinant forms (Luan et al., 2017). In this study, we detected and characterized 2 second generation HIV-1 recombinant strains (BDD034A and BDL060) derived from the CRF01_AE and CRF07_BC subtypes, isolated from MSM infected with HIV-1.

## Materials and methods

In this study, we identified the near full-length genome (NFLG) sequences of two novel HIV-1 URF strains (Sample ID: BDD034A and BDL060) isolated during HIV-1 molecular surveillance in 2022 in Baoding city, Hebei Province, China. The two individuals from which these strains were isolated, BDD034A and BDL060, were a 44-year-old unmarried man and a 45-year-old married man, respectively (see Table 1 for further details). This study was approved by the Medical Ethics Committee of Baoding People’s Hospital (protocol number: 2019–03). Written informed consent was obtained from the subjects prior to sample collection.

**Table 1:** Epidemiological information about the two participants.

Strain name	Sex	Age (years)	Marital status	Transmission route	CD4 T-cell count (cells/μL)	HIV-1 viral load (copies/mL)	Accession number
BDD034A	Male	44	Married	MSM	571	35,200	OP745422
BDL060	Male	45	Married	MSM	379	43,000	OQ207706

RNA was extracted from 140 μL of each subject’s plasma sample using a QIAamp Viral RNA Mini Kit (Qiagen, Duesseldorf, Germany) in accordance with the manufacturer’s instructions. PrimeScript IV 1st Strand cDNA Synthesis Mix (TaKaRa Biotechnology, Dalian, China) was used to reverse transcribe the RNA into 3′ and 5′ half-molecule cDNAs using the primers 1.R3.B3R: 5′-ACTACTTGAAG CACTCAAGGCAAGC TTTATTG-3′ and 07Rev8: 5′-CCTART GGGATGTGTACTT CTG AACTT-3′. A nested polymerase chain reaction (PCR) was performed using TaKaRa Premix Taq (TaKaRa Biotechnology) to amplify the 3′ and 5′ half-molecule regions of the NFLG sequences of BDD034A and BDL060. The reaction conditions and primer sequences used for amplification have been reported previously (Yang et al., 2022). PCR products were detected using 1.0% agarose gel electrophoresis, and amplified products of the expected size were purified from the corresponding electrophoretic bands and sequenced using Sanger sequencing technology by Tianyi Huiyuan Bioscience & Technology Inc. (Beijing, China).

The two NFLG sequences were submitted to the online tool HIV BLAST^2^ to search for similar sequences. Then the two sequences were aligned with subtype reference sequences and CRFs from China^3^ using MAFFT v7.0 (Katoh and Standley, 2013), and the alignments were adjusted manually using BioEdit (v7.2.5.0). Phylogenetic and subregion trees were constructed using MEGA11 (Tamura et al., 2021) with the neighbor-joining (N-J) method (Saitou and Nei, 1987). Recombination breakpoints were identified by SimPlot (v3.5.1) based on Bootscan analyses.

## Results

**Figure 1:**
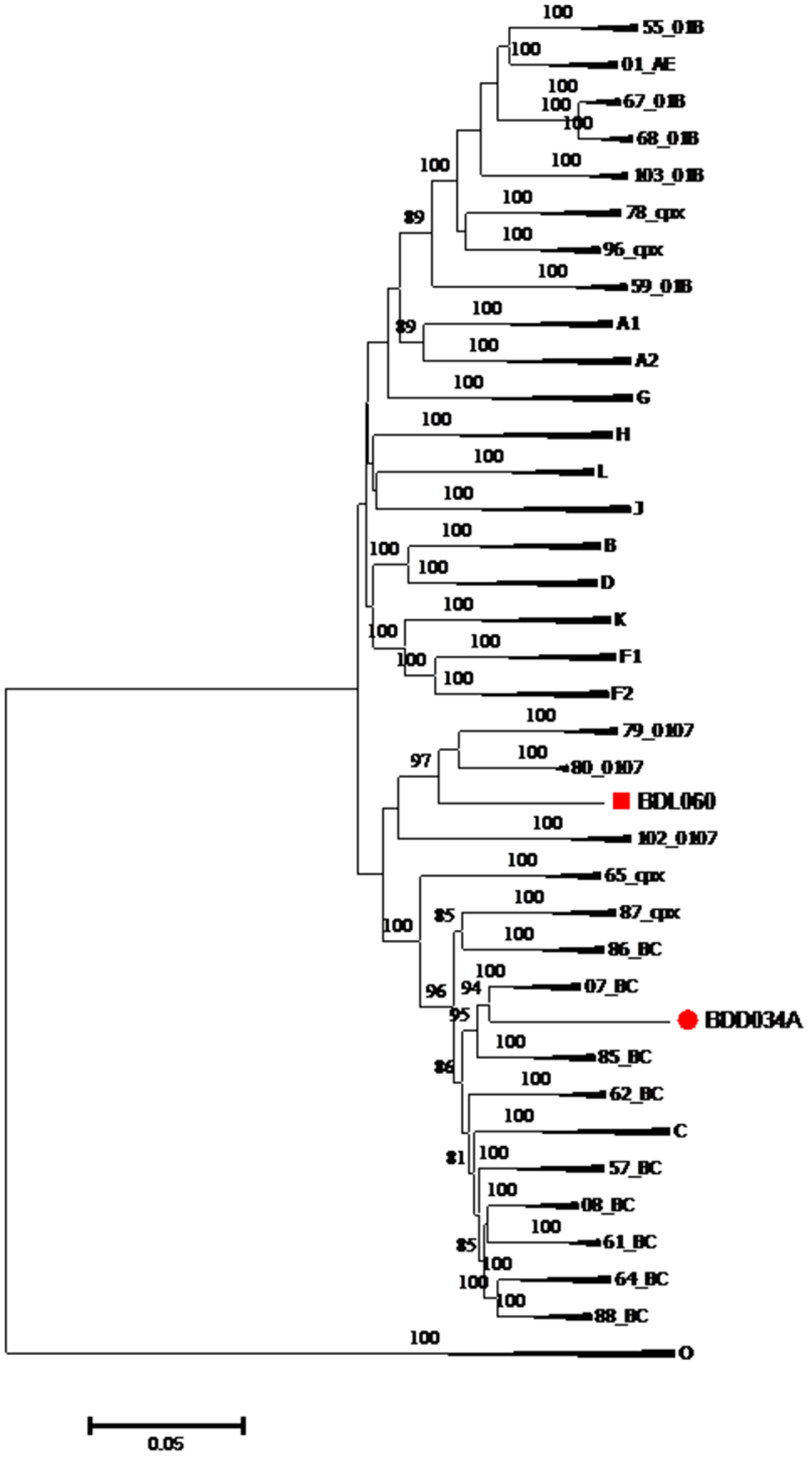
Phylogenetic tree based on the NFLG sequences. The neighbor-joining tree of BDD034A and BDL060 was constructed using Mega 11 with a bootstrap value of 1,000 replicates. Only bootstrap values ≥75% are shown in the corresponding nodes. The scale bar represents a 5% genetic distance.

**Figure 2:**
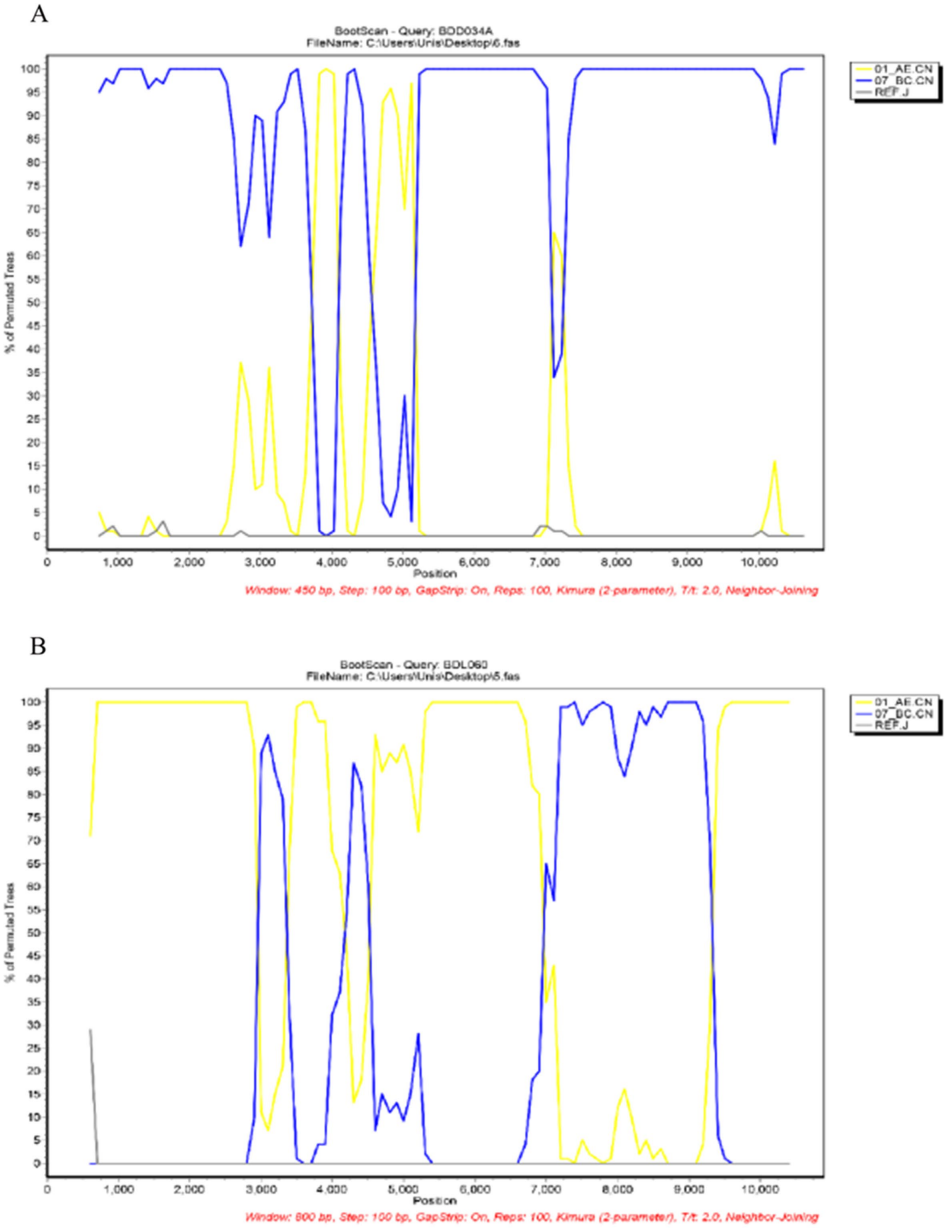
Bootscan analysis. **(A)** Bootscan plot of BDD034A using CRF01_AE (accession numbers JX112801 and JX112859), CRF07_BC (accession numbers HQ215552 and AF286226) and J (accession numbers EF614151 and AF082394) as reference sequences. The parameters were set to a window size of 800 and a step size of 100. **(B)** Bootscan plot of BDL060. The reference sequences were the same as those for BDD034A above. The parameters were set to a window size of 450 and a step size of 100.

**Figure 3:**
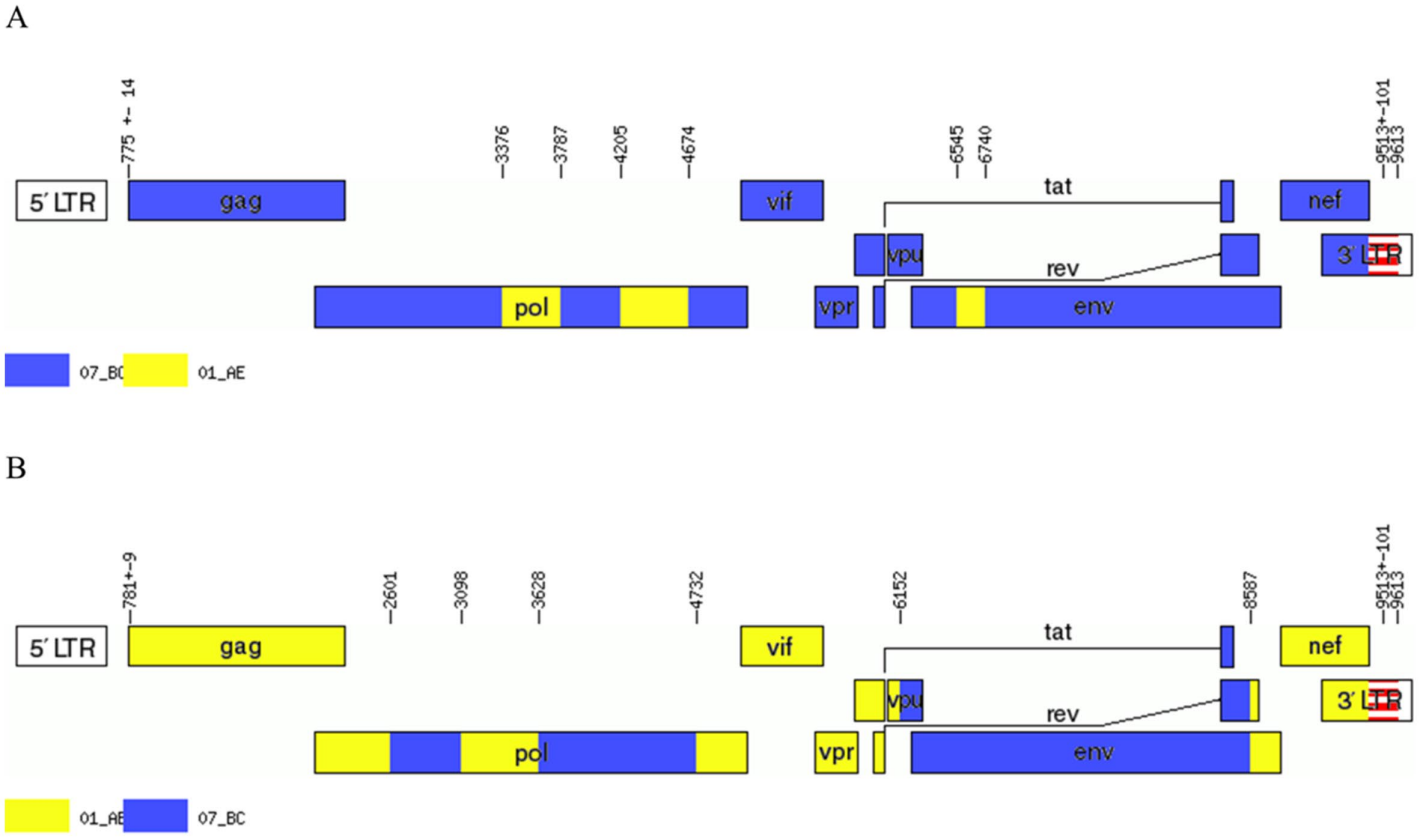
Genetic map of BDD034A **(A)** and BDL060 **(B)**. The Recombinant HIV-1 Drawing Tool was used, which is available at the HIV database: https://www.hiv.lanl.gov/content/sequence/DRAW_CRF/recom_mapper.html.

**Figure 4:**
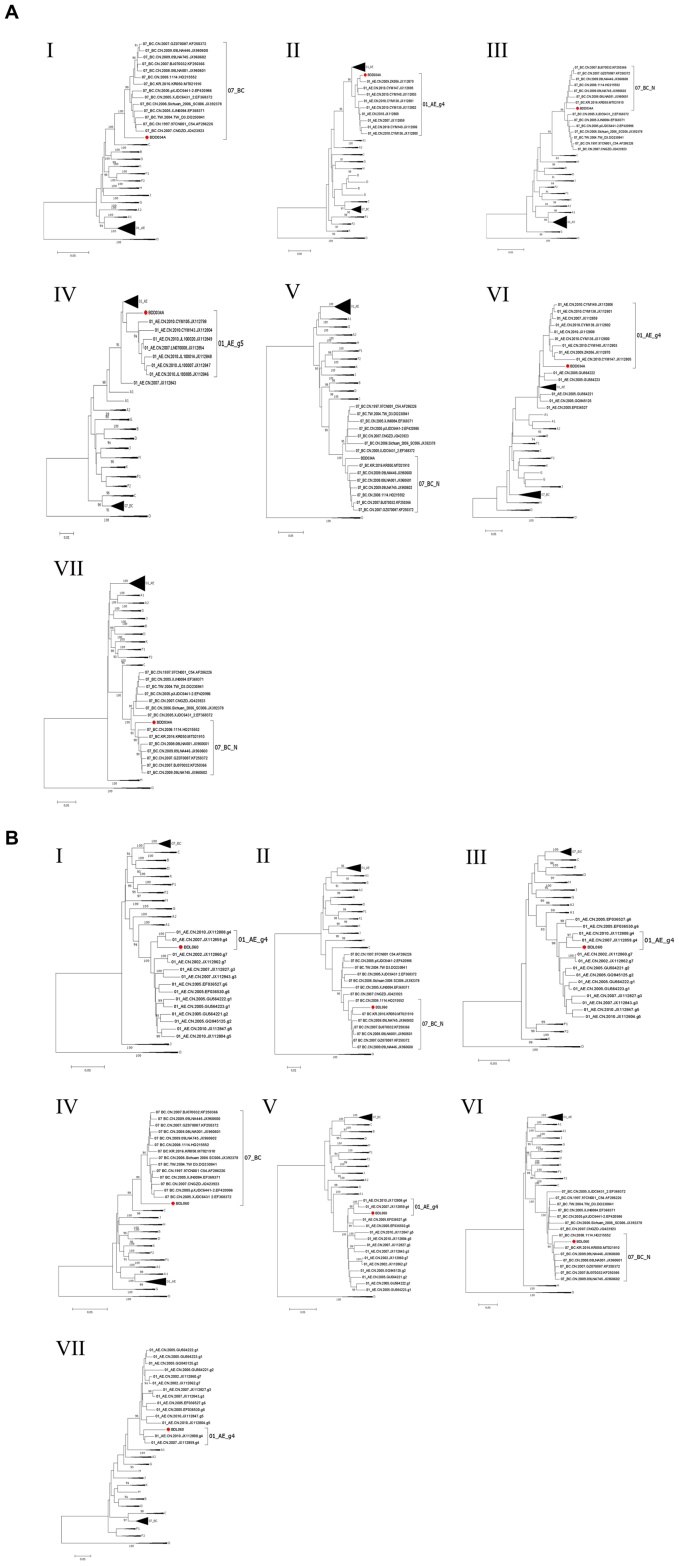
The subregion trees of BDD034A **(A)** and BDL060 **(B)**. The subregion phylogenetic trees were constructed using Mega 11 software and the neighbor-joining method with 1,000 bootstrap replications. Bootstrap values ≥90% are shown at the corresponding nodes. The scale bars represent a 5% genetic distance. Segments of BDD034A and BDL060 are indicated by a solid red circle and a solid red square, respectively.

We acquired two NFLG sequences of 8,890 bp (HXB2: 723–9,613) and 8,847 bp (HXB2: 757–9,604) from BDD034A and BDL060, respectively. The results of HIV BLAST suggested that there are no ≥95% similar sequences found in the HIV database. The constructed NFLG N-J tree showed that both BDD034A and BDL060 formed separate monophyletic branches, indicating that BDD034A and BDL060 are two different novel recombinant forms (Figure 1). The recombinant breakpoint analysis revealed that BDD034A and BDL060 were composed of seven interleaved mosaic gene fragments (Figures 2 3). However, the difference between these sequences was that BDD034A is a combination of three CRF01_AE fragments inserted into the CRF07_BC main framework, whereas BDL060 is a combination of three CRF07_BC fragments inserted into the CRF01_AE main framework (Figure 3A and Figure 3B). The mosaic recombinant structure of the BDD034A sequence can be described as follows: ICRF07_BC (HXB2, 790–3,375 nt); IICRF01_AE (HXB2, 3,376–3,786 nt); IIICRF07_BC (HXB2, 3,787–4,204 nt); IVCRF01_AE (HXB2, 4,205–4,673 nt); VCRF07_BC (HXB2, 4,674–6,544 nt); VICRF01_AE (HXB2, 6,545–6,739 nt); and VIICRF07_BC (HXB2, 6,740–9,411 nt). Subregion phylogenetic analysis was used to confirm the genetic origin of each segment. Segments I, III, V and VII were clustered with CRF07_BC, which is prevalent among MSM in northern China (Figure 4A). Segment II was clustered with CRF01_AE cluster 4 and segment VI, segment IV was clustered with CRF01_AE cluster 5 (Figure 4A). The mosaic recombinant structure of the BDL060 sequence can be described as follows: ICRF01_AE (HXB2, 790–2,600 nt); IICRF07_BC (HXB2, 2,601–3,097 nt); IIICRF01_AE (HXB3, 098–3,627 nt); IVCRF07_BC (HXB3, 628–4,731 nt); VCRF01_AE (HXB2, 4,732–6,151 nt); VICRFF07_BC (HXB2, 6,152–8,587 nt); and VIICRF01_AE (HXB2, 8,588–9,411 nt). Subregion phylogenetic analyses indicated that four CRF01_AE segments mainly originated from CRF01_AE cluster 4 strains among the MSM population in China (Figure 4B).

## Discussion

CRF01_AE is the most important HIV-1 strain in China owing to its significant contribution to the HIV-1 epidemic, and it has been found to possess at least seven gene clusters. CRF01_AE cluster 1 was found primarily among heterosexuals and intravenous drug users (IDUs) in the southern provinces of China. CRF01_AE clusters 1, 2 and 3 were prevalent among heterosexuals and IDUs in southern and southwestern provinces of China. CRF01_AE clusters 4 and 5 were mainly distributed among the MSM population in northern China, including Beijing and Tianjin. Clusters 6 and 7 were only detected among heterosexuals in two southeast and southwest provinces (Feng et al., 2013; Li et al., 2017). In this study, phylogenetic analysis of BDD034A showed that segments II, IV and VI clustered within clusters 4 and 5 of CRF01_AE, respectively. Because BDD034A showed intra-subtype recombination, it does not rule out the possibility of dual infection. CRF07_BC is another dominant HIV-1 strain in China that was originally isolated in 1993 in an IDU in Yunnan Province (Meng et al., 2012), and then spread along drug trafficking routes to Sichuan, Guangxi and Xinjiang Provinces (Tee et al., 2008) in southwestern and northern China. At present, CRF01_AE and CRF07_BC are the predominant intersubtype recombinants among sexually-active populations, especially among MSM in China (Li et al., 2016). High geographical mobility, condom-free sex and multiple sexual partners are contributing factors that lead to an increase in CRFs of HIV-1. The extremely high-risk behaviors and cocirculation of multiple subtypes make MSM significantly more vulnerable to dual infection (Liu et al., 2019). According to our previous report (Shi et al., 2021), the educational level of MSM in Baoding City is low, and they have limited knowledge of HIV prevention. Both of BDD034A and BDL060 have primary education, multiple sexual partners and unprotected anal intercourse. Future research should focus on MSM with low educational level, learn more about HIV prevention, strengthen self-protection, and reduce the risk of HIV transmission. Hebei Province is located in northern China, surrounding Beijing and Tianjin. The convenient transport network in this region enables geographical mobility, and creates opportunities for dual or multiple infections within the MSM population. This has led to the emergence and prevalence of new recombinant strains in MSM in recent years, such as CRF103_01B, CRF123_0107, CRF01_AE/B and CRF01_AE/CRF07_BC (Huang et al., 2019; Zhou et al., 2020; Han et al., 2021; Xing et al., 2021; Fan et al., 2022a,b; Xing et al., 2022). The emergence of new recombinant forms have increased the diversity of HIV-1 isolates prevalent in Hebei province, indicating the further molecular monitoring of HIV-1 diversity is vital in the region.

## Conclusion

In conclusion, we identified two novel recombinant forms of HIV-1 isolated from MSM, which have no similar breakpoints from the CRFs and URFs reported previously. The BLAST search results indicated there are no ≥95% similar sequences in the database with the BDD034A and BDL060 sequences. The emergence of CRF01_AE and CRF07_BC recombinant forms might suggest high genetic variation among HIV-1 in Hebei, warning us to continuously supervise HIV-1 molecular epidemiologic dynamics and gather enough information for vaccine design and to provide effective suggestions for accurate control.

## Data availability statement

The nucleotide sequences of BDD034A and BDL060 have been deposited in the NCBI GenBank database under accession numbers OP745422 and OQ207706, respectively.

## Ethics statement

The studies involving human participants were reviewed and approved by the Medical Ethics Committee at the People’s Hospital of Baoding (2019–03). Written informed consent to participate in this study was provided by the patient/participant or the legal guardian/next of kin of the patient/participant.

## Author contributions

XY, NZ, MS, and WF designed the study. HS, JM, and WA acquired the sequences. JM, JD, MS, and WF analyzed and interpreted the data. XY, NZ, and MS wrote the manuscript. All authors contributed to the article and approved the submitted version.

## Conflict of interest

The authors declare that the research was conducted in the absence of any commercial or financial relationships that could be construed as a potential conflict of interest.

## Publisher’s note

All claims expressed in this article are solely those of the authors and do not necessarily represent those of their affiliated organizations, or those of the publisher, the editors and the reviewers. Any product that may be evaluated in this article, or claim that may be made by its manufacturer, is not guaranteed or endorsed by the publisher.

## Footnotes

^1^ https://www.hiv.lanl.gov/content/sequence/HIV/CRFs/CRFs.html

^2^ https://www.hiv.lanl.gov/content/sequence/BASIC_BLAST/basic_blast.html

^3^ https://hiv.lanl.gov/components/sequence/HIV/search/ search.html
