# Supplementary figures and images for: Characterization of two novel HIV-1 second-generation recombinants (CRF01_AE/CRF07_BC) identified in Hebei Province, China
Source: Front Microbiol. 2023 May 3;14:1159928. doi: 10.3389/fmicb.2023.1159928 (PMC10188989; doi:10.3389/fmicb.2023.1159928)

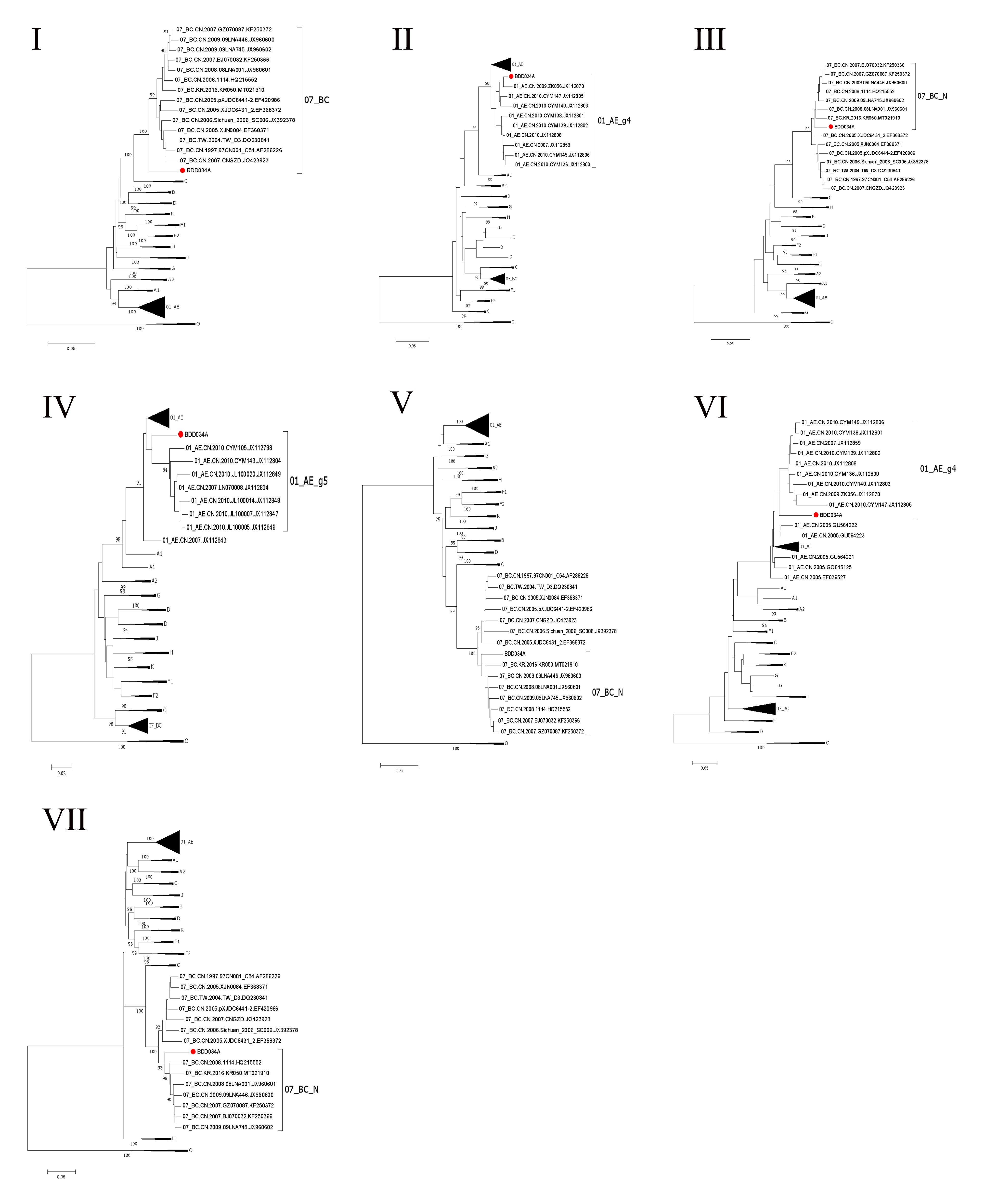

Supplement: Supplementary file 1 [file Image_1.JPEG]

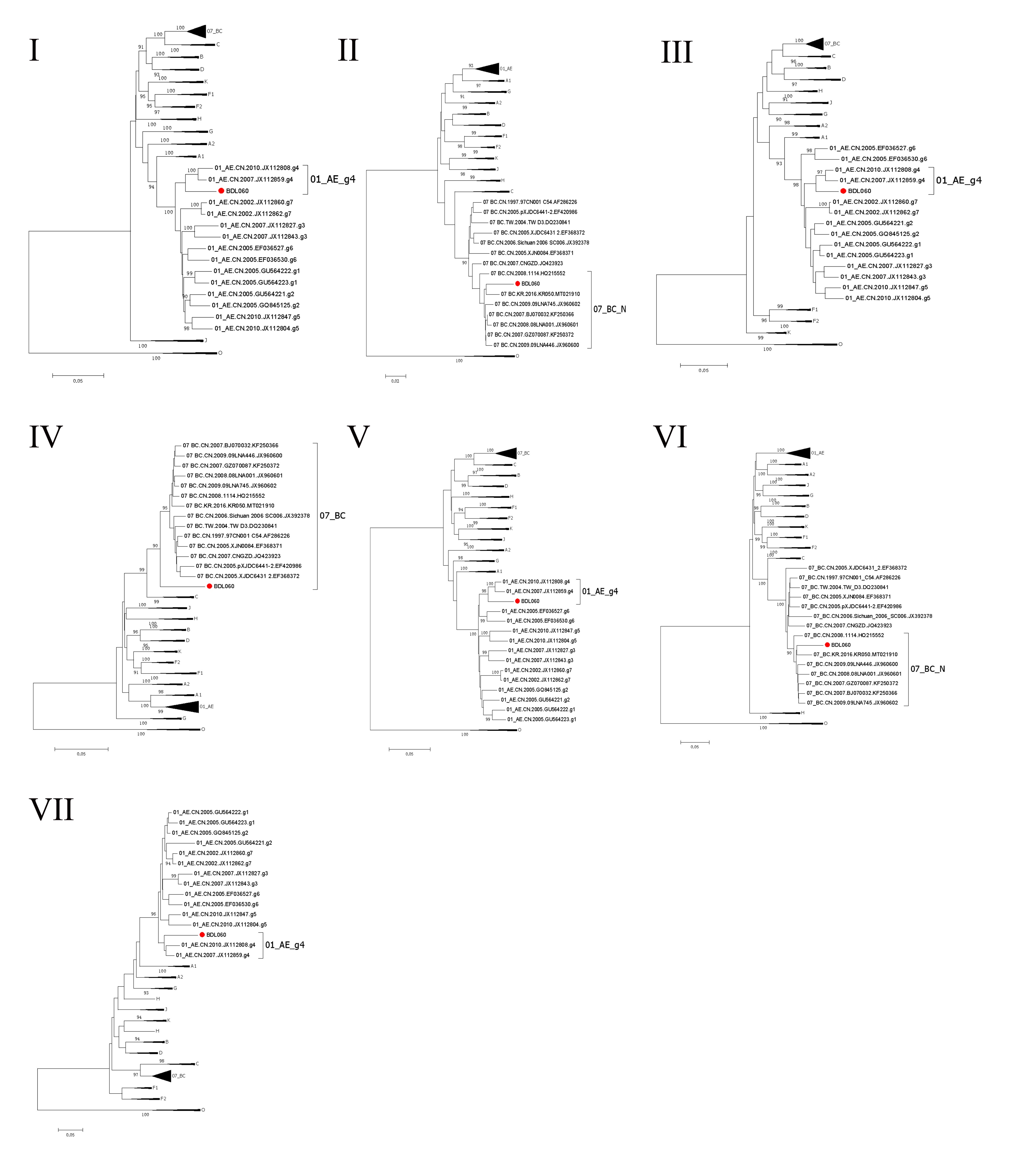

Supplement: Supplementary file 2 [file Image_2.JPEG]
